# Activation of the Unfolded Protein Response Is Required for Defenses against Bacterial Pore-Forming Toxin *In Vivo*

**DOI:** 10.1371/journal.ppat.1000176

**Published:** 2008-10-10

**Authors:** Larry J. Bischof, Cheng-Yuan Kao, Ferdinand C. O. Los, Manuel R. Gonzalez, Zhouxin Shen, Steven P. Briggs, F. Gisou van der Goot, Raffi V. Aroian

**Affiliations:** 1 Section of Cell and Developmental Biology, University of California, San Diego, La Jolla, California, United States of America; 2 Global Health Institute, Ecole Polytechnique Fédérale de Lausanne, Lausanne, Switzerland; Massachusetts General Hospital, United States of America

## Abstract

Pore-forming toxins (PFTs) constitute the single largest class of proteinaceous bacterial virulence factors and are made by many of the most important bacterial pathogens. Host responses to these toxins are complex and poorly understood. We find that the endoplasmic reticulum unfolded protein response (UPR) is activated upon exposure to PFTs both in *Caenorhabditis elegans* and in mammalian cells. Activation of the UPR is protective *in vivo* against PFTs since animals that lack either the *ire-1-xbp-1* or the *atf-6* arms of the UPR are more sensitive to PFT than wild-type animals. The UPR acts directly in the cells targeted by the PFT. Loss of the UPR leads to a normal response against unrelated toxins or a pathogenic bacterium, indicating its PFT-protective role is specific. The p38 mitogen-activated protein (MAPK) kinase pathway has been previously shown to be important for cellular defenses against PFTs. We find here that the UPR is one of the key downstream targets of the p38 MAPK pathway in response to PFT since loss of a functional p38 MAPK pathway leads to a failure of PFT to properly activate the *ire-1-xbp-1* arm of the UPR. The UPR-mediated activation and response to PFTs is distinct from the canonical UPR-mediated response to unfolded proteins both in terms of its activation and functional sensitivities. These data demonstrate that the UPR, a fundamental intracellular pathway, can operate in intrinsic cellular defenses against bacterial attack.

## Author Summary

Pore-forming toxins (PFTs) are bacterial toxins that form holes at the plasma membrane of cells and play an important role in the pathogenesis of many important human pathogens. Although PFTs comprise an important and the single largest class of bacterial protein virulence factors, how cells respond to these toxins has been understudied. We describe here the surprising discovery that a fundamental pathway of eukaryotic cell biology, the endoplasmic reticulum unfolded protein response (UPR), is activated by pore-forming toxins in *Caenorhabditis elegans* and mammalian cells. We find that this activation is functionally important since loss of either of two of the three arms of UPR leads to hypersensitivity of the nematode to attack by PFTs. The response of the UPR to PFTs can be separated from its response to unfolded proteins both at the level of activation and functional relevance. The response of the UPR to PFTs is dependent on a central pathway of cellular immunity, the p38 MAPK pathway. Our data show that the response of cells to bacterial attack can reveal unanticipated uses and connections between fundamental cell biological pathways.

## Introduction

Pore-forming toxins (PFTs) are the single most prevalent protein virulence factor made by disease-causing bacteria and are important for the virulence of many important human pathogens including *Staphylococcus aureus*, *Streptococcus pyogenes*, *Clostridium perfringens*, and *Aeromonas hydrophilia* [1],[2]. Crystal (Cry) toxins produced by the invertebrate pathogen *Bacillus thuringiensis* (Bt) are a large family of PFTs that target the intestinal cells of insects and nematodes [3],[4],[5]. The fact that some Cry proteins target nematodes, in particular *C. elegans*, has been exploited to provide the only *in vivo* genetic model for studying PFTs. This system led to the discovery of the first signal transduction pathway that protects cells against PFTs, the p38 mitogen-activated protein kinase (MAPK) pathway, which has been confirmed in mammalian cells [6],[7]. There is growing evidence that the response of cells to PFTs is, however, complex and there is a great deal yet to learn [8].

The unfolded protein response (UPR) of the endoplasmic reticulum (ER) is a fundamental stress response used by eukaryotic cells to match protein synthesis demand to its capability to fold proteins within the ER to maintain cellular homeostasis [9]. In *C. elegans* and other animals there are three transducers that signal from the ER to activate this response. These three distinct arms of the UPR are mediated by IREI, ATF6, and PERK in mammals [10], which correspond to the genes *ire-1, atf-6*, *and pek-1* in *C. elegans* [11],[12],[13]. All three pathways are regulated by the ER chaperone BiP in response to an increase in unfolded proteins [9].

Here we demonstrate that the ER stress response, in particular the *ire-1* arm, is activated upon exposure of *C. elegans* and mammalian cells to PFTs. We demonstrate for the first time that the *ire-1 – xbp-1* arm of the UPR (and to a lesser extent the *atf-6* arm) is functionally important for defense against a pathogenic attack since loss of this pathway leads to animals hypersensitive to PFT, but not to other toxic insults. Furthermore, we demonstrate that activation of the *ire-1-xbp-1* pathway by PFT requires p38 MAPK and its associated MAPK kinase and that the *in vivo* response of the UPR to a PFT can be separated from its response to unfolded proteins. These results indicate that activation of the UPR plays an important role in cellular defenses against pathogens.

## Results

### Cry5B activates the IRE-1 UPR pathway

**Figure 1:**
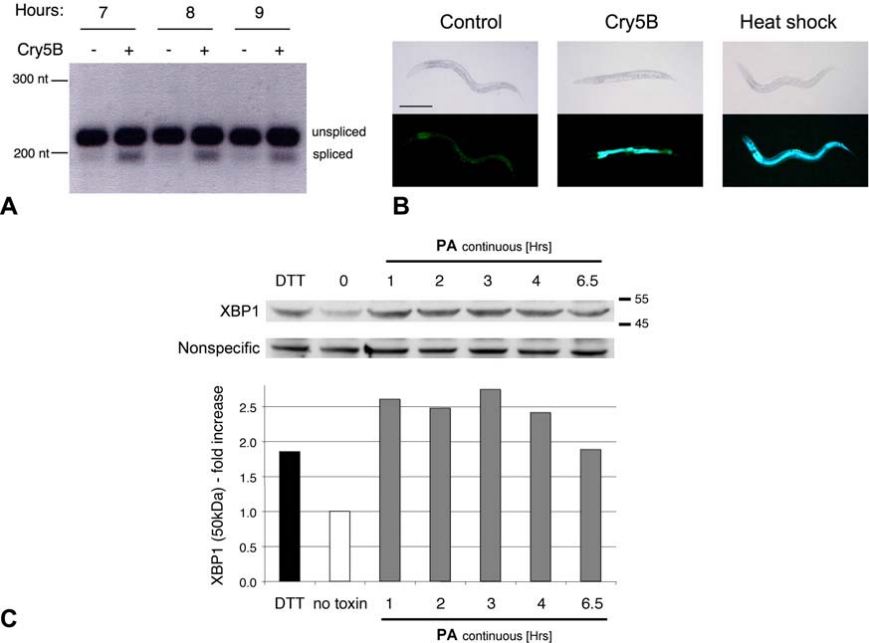
The IRE-1 UPR is activated in response to PFTs. (A) *xbp-1* mRNA splicing is induced in wild-type *C. elegans* fed *E. coli* expressing Cry5B compared to control *E. coli* not expressing Cry5B. The time the worms were allowed to feed on the *E. coli* before total RNA was prepared for RT-PCR is indicated at the top, and the positions of the nucleotide size markers are indicated at the left. (B) Compared to worms fed control non-Cry5B expressing *E.* coli, *in vivo* activation of *hsp-4::GFP* occurs specifically in the intestines of worms fed Cry5B expressing *E. coli* at 20°C for 8 hours. As a comparison for GFP induction, separate worms on control bacteria were heat shocked at 30°C for 8 hours to induce the ER stress response by causing unfolded proteins. The heat shock worms have a strong increase in GFP throughout the body including the head, intestine and hypodermis. Thus, although the entire worm is capable of activating the *ire-1*-*xbp-1* pathway as judged by *hsp-4* induction, activation in Cry5B-fed animals is occurring only in those cells targeted by the PFT. Images taken by light microscopy are compared to images with fluorescence microscopy. Scale bar is 0.2 mm. The experiment was performed three times, and representative worms are shown. (C) Aerolysin induces activation of IRE1 in mammalians cells. Exposure of HeLa cells to proaerolysin (2 ng/mL) leads to increased production of spliced XBP1 protein as shown on this immunoblot (upper) and quantitated relative to no toxin control (lower). DTT (10 µg/mL for 2 h) was used as a positive control. Positions of molecular weight markers (kDa) are indicated on right side of the figure. A nonspecific antibody-reacting band was used as a loading control and normalization of the XBP1 signal in each lane.

In a genetic screen for genes involved in the cellular response of *C. elegans* to the PFT Cry5B, we found a mutant predicted to be defective in protein N-glycosylation in the ER (L.J.B. and R.V.A., manuscript in preparation). Since defects in protein glycosylation induce the UPR, this result suggested that perhaps the UPR might play a role in protection against PFTs. To test this hypothesis, we first investigated whether or not the UPR was activated by a PFT. The *xbp-1* gene is spliced upon activation of the IRE-1 branch of the UPR, and its splicing is one marker for IRE-1 (and UPR) activation [13]. In *C. elegans*, the *xbp-1* intron spliced by IRE-1 is 23 nucleotides and the induction of this splicing event can be detected by RT-PCR [14]. To analyze *xbp-1* mRNA transcript splicing, animals were fed *Escherichia coli* expressing Cry5B and compared to worms fed control *E. coli* (Figure 1A). While there is abundant unspliced *xbp-1* mRNA transcript in both samples, there is an increase in the spliced *xbp-1* transcript from worms ingesting Cry5B, indicating activation of the IRE-1 pathway. Quantitative analyses indicate that the *xbp-1* spliced transcript increases 2.3, 3.0, and 3.0 fold at the 7, 8, and 9 h time points respectively.

To independently test this result, we analyzed the *in vivo* expression of an *ire-1* regulated gene, *hsp-4*, a BiP homolog. *In vivo* analysis of the *hsp-4* promoter coupled to green fluorescent protein (GFP) demonstrated expression of this gene requires *ire-1* and *xbp-1* [13]. A *C. elegans* strain containing *hsp-4::GFP* was fed either control *E. coli* or Cry5B expressing *E. coli* for 8 hours at 20°C. As shown, a strong and specific increase in GFP expression in the intestine can be seen in the presence of the PFT (Figure 1B, middle panel), consistent with activation of the *ire-1-xbp-1* pathway by Cry5B. Heat shock of this strain in the absence of Cry5B confirms GFP could be induced in other cell types in addition to the intestine (Figure 1B, right panel), as was demonstrated with the N-glycosylation inhibitor tunicamycin [13]. The fact that Cry5B only induced expression in intestinal cells suggests the PFT is only targeting these cells (see below).

To address whether the *ire-1*-*xbp-1* pathway is also activated in mammalian cells in response to a PFT, activation of the pathway was ascertained in HeLa cells exposed to the *Aeromonas hydrophila* PFT, aerolysin. As detected by the presence of the spliced protein isoform of XBP-1, treatment of mammalian cells with a PFT also results in robust activation of the *ire-1*-*xbp-1* pathway (Figure 1C).

### The ER stress response is required for defense of *C. elegans* against Cry5B

**Figure 2:**
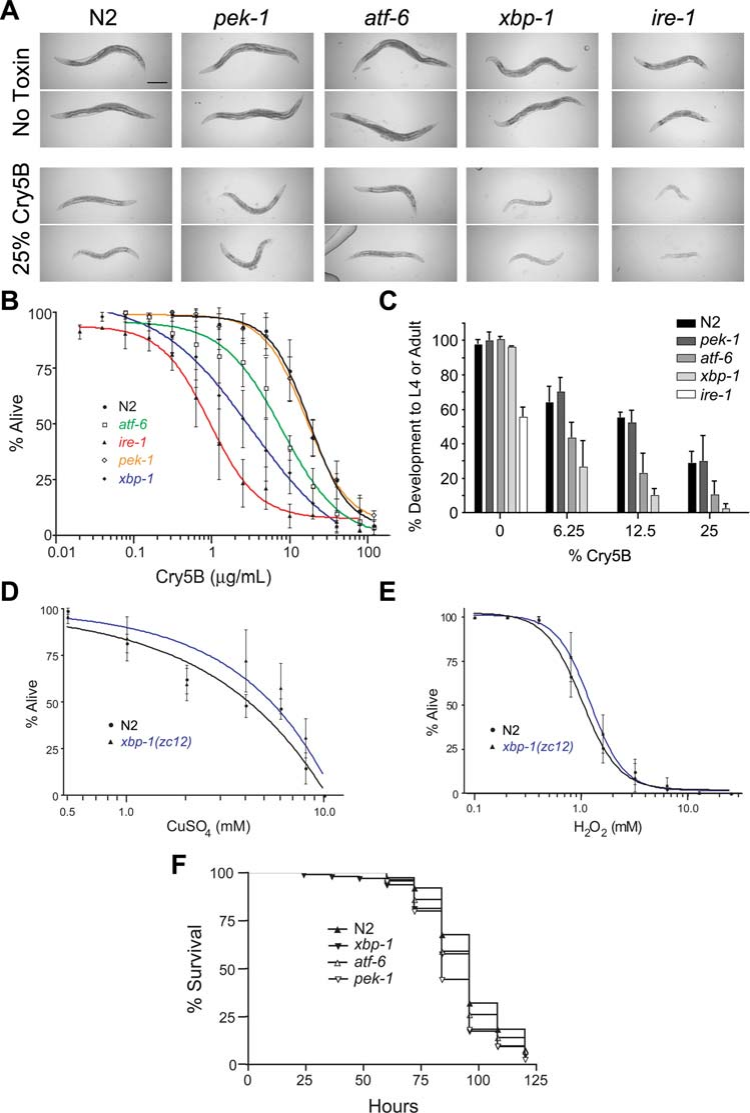
Loss of specific UPR pathways cause hypersensitivity to PFT but not other toxins or a pathogenic bacteria. (A) Comparison of ER stress response mutants to wild-type N2 on 25% Cry5B-expressing *E. coli* plates indicate *ire-1(v33)* and *xbp-1(zc12)* are hypersensitive to Cry5B intoxication. Two representative worms are shown for each strain 48 hours after feeding either on *E. coli* without Cry5B or on *E. coli* of which 25% expressed Cry5B. Scale bar is 0.2 mm. (B) A lethal concentration assay was performed using purified Cry5B toxin to quantitatively compare sensitivities of wild-type N2 and the ER stress mutants. Lethality was determined after 8 days. This semi-log graph represents three independent experiments, and each data point is the mean and standard deviations of the experiments. (C) A Cry5B developmental inhibition assay was performed beginning with synchronized worms at the first larval stage. Worms were grown on plates containing different percentages of Cry5B-expressing *E. coli* (% Cry5B as indicated under the figure), and the percent of worms reaching the L4 stage or adulthood 72 hours later is indicated. *ire-1(v33)* was included only on the plates with 0% Cry5B. Data are presented as mean and standard deviation. (D) A lethal concentration assay comparing sensitivity to CuSO_4_ revealed *xbp-1(zc12)* is not hypersensitive compared to wild-type N2. Lethality was determined after 8 days of CuSO_4_ exposure, the same time frame as the Cry5B lethality assay. Data, plotted semi-log, are the mean and standard deviation of three independent experiments. (E) A lethal concentration assay comparing sensitivity to H_2_O_2_ revealed *xbp-1(zc12)* is not hypersensitive compared to wild-type N2. Lethality was determined after 4 hours of H_2_O_2_ exposure. Data, plotted semi-log, are the mean and standard deviation of three independent experiments. (F) A lifespan assay was used to compare the ER stress mutants to slow killing by *P. aeruginosa PA14*. This graph represents combined data from three experiments.

To determine whether the ER stress response played a role in the defense of *C. elegans* against the PFT, *C. elegans* mutants in the ER stress response pathway were qualitatively compared to wild-type N2 animals in their susceptibilities to Cry5B. The mutants that were tested included those encoding the three ER stress transducer genes, *atf-6(ok551)*, *pek-1(ok275)*, and *ire-1(v33)*, as well as *xbp-1(zc12)*; these mutations are predicted or known to be loss of function mutations in their respective genes [11],[12],[13]. In the absence of Cry5B, the wild type and mutant worms are healthy adults with similar appearance, except *ire-1(v33)*, which is clearly smaller than the other strains (Figure 2A). In the presence of low-moderate levels of the PFT Cry5B, wild-type worms are slightly intoxicated compared to those found on control no-toxin plates, as evidenced by their smaller sizes and paler appearances (Figure 2A). To the same extent seen with wild-type worms, *atf-6(ok551)* and *pek-1(ok275)* mutant animals are also slightly intoxicated on low-moderate levels of the PFT Cry5B, indicating lack of either of these genes does not result in overt hypersensitivity or hyper-resistance to Cry5B (Figure 2A). However under the same conditions, the *ire-1(v33)* and *xbp-1(zc12)* mutant worms are more severely intoxicated than wild-type worms as they are relatively smaller and considerably paler compared to their corresponding no toxin controls. The hypersensitivity to Cry5B resulting from lack of *ire-1* and *xbp-1* was also seen using RNA interference (RNAi; data not shown), confirming the phenotype is caused by loss of function in these genes. We call this hypersensitivity phenotype “Hpo” for ***h***ypersensitive to ***po***re-forming toxin.

The sensitivity to Cry5B of animals mutant for the three ER stress response pathways was quantitatively assessed using a dose-dependent lethality assay (Figure 2B). From these data, an LC_5s_s (lethal concentrations at which 50% of the animals die) were obtained (Table 1). Our quantitative results confirm that *ire-1(v33)* and *xbp-1(zc12)* mutant animals are statistically more sensitive to PFT than wild type animals (Table 1) and thus are Hpo relative to wild type (caution is called for in interpreting the *ire-1(v33)* data since many of these animals also have significant overt defects, *e.g.*, developmental delays which prevents them from being as well synchronized at the start of the assay compared to the other strains [11]). Our results indicate that *atf-6(ok551)* mutant animals are also Hpo, albeit to a lesser extent (2.8 vs. 5.8 fold increase in sensitivity for *atf-6* vs. *xbp-1*). Although *atf-6(ok551)* hypersensitivity was not discerned with the plate assay, it is likely that the quantitative lethality assay is a more sensitive test for Cry5B hypersensitivity than the qualitative plate assay. In contrast to *xbp-1* and *atf-6* mutant animals, the sensitivity of *pek-1(ok275)* mutant animals is not statistically different from that of wild-type animals (Table 1).

**Table 1:** Data analysis of the Cry5B, CuSO_4_ and H_2_O_2_ lethal concentration assays and *P. aeruginosa* (PA14) lifespan assay.

Cry5B				
Strain	LC_50_ (µg/mL)	Standard Deviation	p value	Relative Sensitivity (LC_50_ wild type/LC_50_ mutant)
Wild type (N2)	18.6	5.76		
*pek-1(ok275)*	18.3	4.79	>0.05	1.02
*atf-6(ok551)*	6.70	2.53	<0.01	2.78
*xbp-1(zc12)*	3.22	1.72	<0.001	5.78
*ire-1(v33)*	1.40	0.59	<0.001	13.3

CuSO_4_			
Strain	LC_50_ (mM)	Standard Deviation	p value
Wild type (N2)	3.26	0.19	
*xbp-1(zc12)*	4.16	1.07	0.34

H_2_O_2_			
Strain	LC_50_ (mM)	Standard Deviation	p value
Wild type (N2)	1.20	0.21	
*xbp-1(zc12)*	1.38	0.38	0.87

PA14	
Strain	Median Survival (Hrs)
Wild type (N2)	96
*pek-1(ok275)*	84
*atf-6(ok551)*	96
*xbp-1(zc12)*	96

Cry5B				
Strain	LC_50_ (µg/mL)	Standard Deviation	p value	Relative Sensitivity (LC_50_ wild type/LC_50_ mutant)
Wild type (N2)	20.4	12.3		
*sek-1(km4)*	0.12	0.054	<0.05	170

The p value for comparison of the PA14 survival curves was p = 0.05.

To independently confirm these results, we used a developmental assay to assess the relative sensitivity of the four ER stress response mutants to Cry5B. This experiment was performed by placing newly hatched L1 stage worms on plates containing different percentages of Cry5B expressing *E. coli* and then counting the worms that developed to either the L4 stage or adulthood (Figure 2C). In the absence of Cry5B, nearly all worms developed to the L4 stage or adulthood for all strains with the exception of *ire-1(v33)*. This result confirms developmental defects previously seen with this mutant [11], and it was therefore excluded from subsequent analyses. Wild type N2 and *pek-1(ok275)* were both similarly inhibited in their development by increasing percentages of Cry5B. Compared to N2 and *pek-1(ok275)* animals, though, both *atf-6(ok551)* and *xbp-1(zc12)* were Hpo, *i.e.*, each is more developmentally inhibited by Cry5B than wild-type animals (Figure 2C). Because *the ire-1-xbp-1* pathway has a more discernible effect on protection against Cry5B than *atf-6*, further experiments were focused on this arm of the ER stress response.

Taken together, the above results suggest that the *ire-1-xbp-1* pathway functions to protect the host against the PFT Cry5B. However, an alternative explanation for our results is that animals mutant in this pathway (*e.g.*, *xbp-1* mutant animals) are sickly and have compromised health and therefore would respond poorly to any toxic insult. To address this alternative hypothesis, we tested whether *xbp-1(zc12)* animals are hypersensitive to two toxic chemical compounds, the heavy metal CuSO_4_ (a toxic insult that kills with kinetics similar to Cry5B) and the oxidative stress agent H_2_O_2_ (a toxic insult that kills rapidly). The mutant *xbp-1(zc12)* has the same sensitivity as wild type to killing by either CuSO4 or H_2_O_2_ (Figure 2D and 2E; Table 1). These data argue against the supposition that this mutant is hypersensitive to the PFT merely because it is generally unhealthy. Rather, the protective response is somewhat specific against the PFT. These conclusions are strengthened by the finding that *C. elegans* lacking the UPR respond normally to attack by the pathogenic bacteria *Pseudomonas aeruginosa*, which does not make a PFT (Figure 2F and Table 1).

### The *xbp-1* pathway functions in the intestine to protect against Cry5B PFT

Mosaic and expression analyses have shown that the targeting of intestinal cells by the PFT Cry5B is both necessary and sufficient to intoxicate worms [15],[16]. If the *ire-1-xbp-1* pathway is functioning directly to protect against the effects of the PFT, then we would predict that the *ire-1-xbp-1* pathway should function in the target cells of the toxin, the intestinal epithelial cells. Alternatively, the pathway might be functioning indirectly to protect against the effects of the PFT (*e.g.*, it might hypothetically function in neurons that then sends protective signals to the intestine). Consistent with the first hypothesis, that the pathway is functioning directly in the target cells to protect against the PFT, we previously noted that a marker for downstream activation of the pathway, *hsp-4*, is turned on exclusively in intestinal cells (Figure 1B, middle panel), although the pathway is capable of being activated throughout the worm by a more general stress, such as heat shock (Figure 1B, right panel).

**Figure 3:**
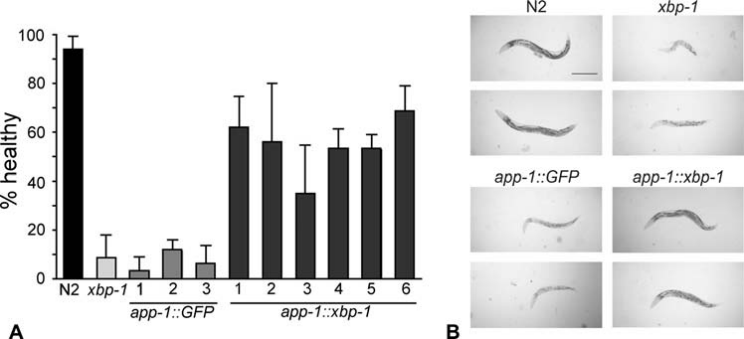
Intestinal specific expression of *xbp-1* is sufficient to rescue sensitivity to the PFT. Sensitivity to Cry5B was compared among wild-type N2, *xbp-1(zc12)*, *xbp-1(zc12)* transformed with *app-1::GFP*, and *xbp-1(zc12)* transformed with *app-1::xbp-1* animals using a plate feeding assay. (A) The health of the worms (details in Materials and Methods) was evaluated after 72 hours on 25% Cry5B-expressing *E. coli*. Three and six independent lines of *app-1::GFP* and *app-1::xbp-1* were used, respectively. Data are mean and standard deviation of three experiments. (B) Images comparing the health of wild-type N2, *xbp-1(zc12)*, *xbp-1(zc-12) app-1::GFP*, and *xbp-1(zc-12) app-1::xbp-1* animals on 25% Cry5B plates for 72 h. Scale bar is 0.2 mm.

To directly demonstrate the role of *xbp-1* in protecting intestinal cells against Cry5B, the intestinal specific *app-1* promoter [17] was used to drive expression of *xbp-1* in *xbp-1(zc12)* mutant animals to determine if expression in the intestine is sufficient to rescue the Hpo phenotype. As a negative control, GFP was similarly expressed under control of the *app-1* promoter in *xbp-1(zc12)* mutant animals. In control animals, expression of the GFP solely in intestinal cells was confirmed (data not shown). As expected, the majority of wild-type N2 animals showed only a low-modest degree of intoxication upon exposure to 25% Cry5B-expressing *E. coli* (Figure 3A, B; they were smaller and somewhat paler than the wild-type worms on control plates but were still quite active). Also as predicted, both *xbp-1(zc12)* mutant animals and *xbp-1(zc12)* mutant animals transformed with *app-1::GFP* were Hpo and intoxicated to similar extents (Figure 3A, B; most animals were very pale, small, and inactive). In contrast, *xbp-1(zc12)* worms expressing *xbp-1* under the *app-1* promoter were significantly healthier than either untransformed or *app-1::GFP* transformed *xbp-1(zc12)* animals fed with Cry5B (Figure 3A, B). However, these *app-1::xbp-1*-transformed *xbp-1(z12)* worms were not as healthy as wild-type N2 under the same conditions. This partial rescue could indicate the expression of the artificial *xbp-1* transgenes did not fully recapitulate wild-type *xbp-1* expression levels and/or that there is some role for the *ire-1* – *xbp-1* pathway in other cell types. Nonetheless, our results support a significant protective function for *xbp-1* within the cells targeted by Cry5B.

### Induction of *ire-1-xbp-1* pathway's role in response to PFT but not unfolded proteins is regulated by the p38 MAPK pathway

**Figure 4:**
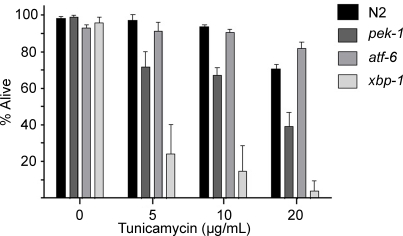
The ER stress response mutants differ in their sensitivities to tunicamycin. A lethality assay was used to compare sensitivities of the ER stress mutants and wild-type N2 to tunicamycin. The percent of worms alive after 8 days of exposure to each concentration of tunicamycin was determined. Data are the mean and standard deviation of three independent experiments.

ER stress responses have been studied extensively for their role in protecting cells against unfolded proteins [10],[18]. One way to assess the role of the ER stress pathways in protecting against unfolded proteins is with the drug tunicamycin (a natural compound that leads to the accumulation of unfolded proteins in the ER due to its inhibitory effect on N-linked protein glycosylation [19]). Previous data in *C. elegans* have indicated different sensitivities of the three ER stress response pathways for tunicamycin [11],[12]. Using a different toxicity assay, we have confirmed these observations: *atf-6(ok551)* mutant animals have a similar sensitivity to tunicamycin as wild-type animals whereas both *xbp-1(zc12)* and *pek-1(ok275)* mutant animals are more readily killed by tunicamycin (Figure 4). These results are in contrast to the response of these different ER stress pathways to Cry5B, to which *atf-6* mutant animals are more sensitive than *pek-1* mutant animals. These data suggest that there are differences in how ER stress pathways are activated in response to unfolded proteins and to the PFT Cry5B.

It is known that PFTs trigger the activation of p38 MAPK, which promotes cell survival and cellular defenses and which seems to play a central role in cellular responses to PFTs [6],[7],[20]. We therefore investigated whether PFT-mediated activation of the UPR and the p38 MAPK pathway might be connected.

**Figure 5:**
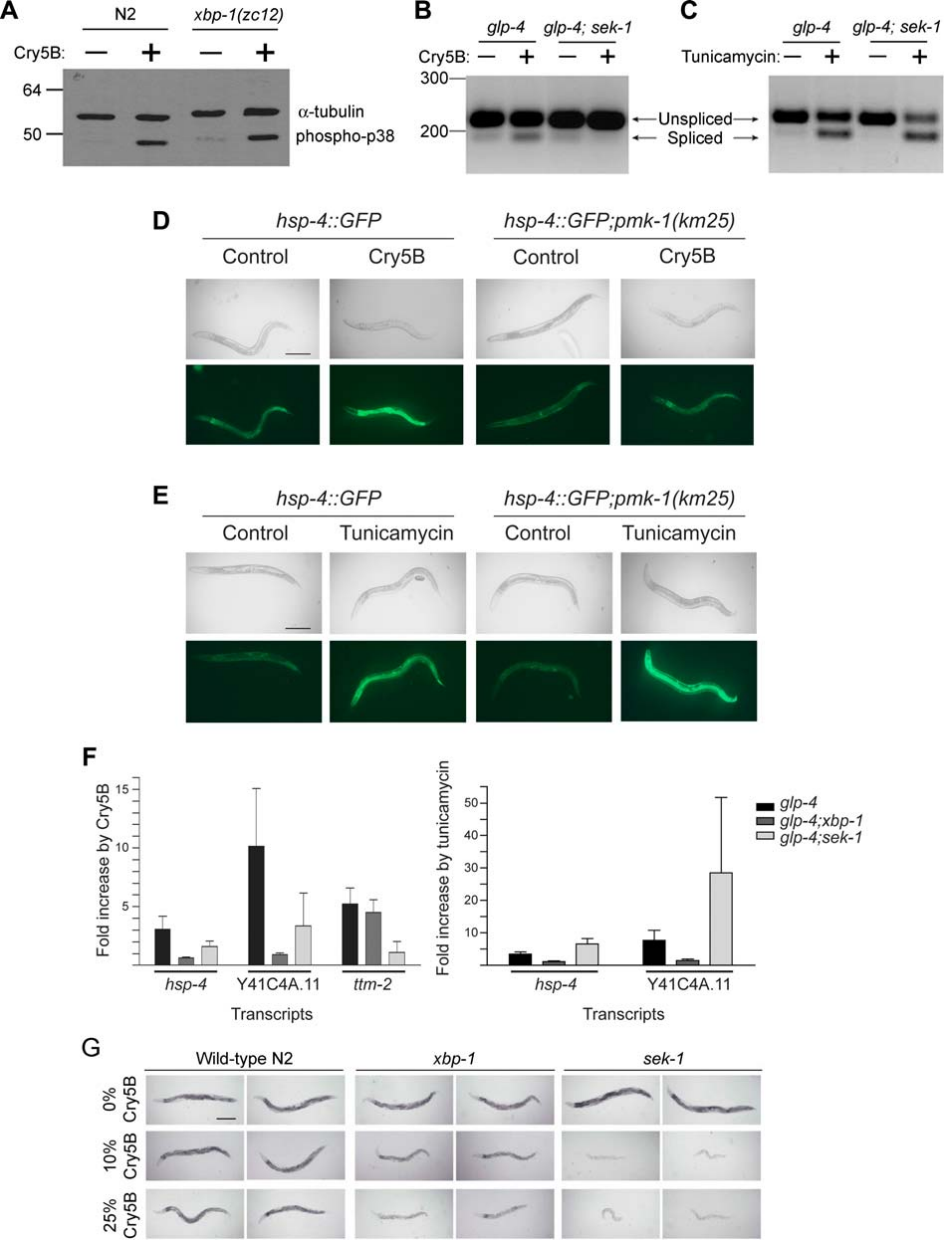
Relationship of the p38 MAPK and UPR pathways in response to PFT and unfolded proteins. (A) The *xbp-1* pathway is not required for phosphorylation of p38 MAPK by Cry5B. Wild-type N2 and *xbp-1(zc12)* were exposed to either control buffer or purified Cry5B toxin for one hour. Worm lysates were analyzed by immunoblotting for phospho P38 MAPK along with α-tubulin as a loading comparison. Positions of molecular weight markers in kilodaltons are shown on left side of gel. Data are representative of three independent experiments. (B) Cry5B induced splicing of *xbp-1* requires *sek-1* (MAPKK). Splicing of *xbp-1* mRNA was compared in *glp-4(bn2)* and *glp-4(bn2);sek-1(km4)* after 3 hours of exposure to either control *E. coli* or *E. coli* expressing Cry5B. Size markers in nucleotides are indicated on the left. This is a representative experiment of three independent experiments. (C) Tunicamycin induced splicing of *xbp-1* does not require *sek-1* (MAPKK). Splicing of *xbp-1* mRNA was compared in *glp-4(bn2)* and *glp-4(bn2)*;*sek-1(km4)* after 3 hours of exposure to either control (DMSO) or tunicamycin (2 µg/mL). This is a representative experiment of three independent experiments. (D) *In vivo* induction of *hsp-4::GFP* by Cry5B requires *pmk-1* (p38 MAPK). The strains *hsp-4::GFP* and *hsp-4::GFP*;*pmk-1(km25)* were fed either control *E. coli* or *E. coli* expressing Cry5B for 8 hours and the expression of GFP was then analyzed. Cry5B induces GFP within the intestinal cells of the strain *hsp-4::GFP* but not in the strain containing the *pmk-1(km25)* mutant. The experiment was performed three times and representative worms are shown. Scale bar is 0.2 mm. (E) *In vivo* induction of *hsp-4::GFP* by tunicamycin does not require *pmk-1* (p38 MAPK). The strains *hsp-4::GFP* and *hsp-4::GFP*;*pmk-1(km25)* were exposed to either control (DMSO) or tunicamycin (2 µg/mL) for 8 hours and the expression of GFP was then analyzed. Tunicamycin induces GFP throughout both the strains *hsp-4::GFP* and *hsp-4::GFP;pmk-1(km25)*, including within the intestinal cells. The experiment was performed three times and representative worms are shown. Scale bar is 0.2 mm. (F) Downstream targets of the UPR require the p38 MAPK pathway for induction by PFT but not unfolded proteins. The fold change in the levels of *hsp-4* and Y41C4A.11 mRNA transcripts by Cry5B and tunicamycin were determined for *glp-4(bn2)*, *glp-4(bn2)*;*xbp-1(zc12)* and *glp-4(bn2)*;*sek-1(km4)* using real-time PCR. In addition, the fold change in *ttm-2* transcripts was determined in response to Cry5B. Data are mean and standard deviation of three independent experiments. (G) Animals lacking *sek-1* MAPKK are more sensitive to Cry5B than animals lacking *xbp-1*. Wild-type N2, *sek-1(km4)*, and *xbp-1(zc12)* animals were placed on plates spread with *E. coli* transformed with empty vector (0%) or spread with empty vector *E. coli* diluted 9∶1 (10%) or 3∶1 (25%) with Cry5B-expressing *E. coli* (% thus gives toxin dose on a plate relative to undiluted Cry5B-expressing *E. coli*). The assay was initiated with L4 stage worms and photographs were taken 48 hours later. In the absence of Cry5B, the worms developed into dark, gravid, active, healthy adults. On 10% Cry5B-expressing *E. coli*, *xbp-1(zc12)* were slightly smaller than N2 but healthier than *sek-1(km4)*, which were as small, pale, inactive, and severely intoxicated. On 25% Cry5B-expressing *E. coli*, *xbp-1(zc12)* was more intoxicated than N2 but not as intoxicated as *sek-1(km4)* animals. Scale bar is 0.2 mm.

We first investigated whether the *ire-1-xbp-1* pathway plays a role in the PFT-induced activation of p38 by comparing the activation of the p38 MAPK in wild-type and *xbp1(zc12)* animals. We find that addition of Cry5B to wild-type *C. elegans* results in an increase in phosphorylated p38, indicating the p38 pathway is activated by a PFT in *C. elegans* just as it is in mammalian cells [20] (Figure 5A). We find that p38 activation occurs normally in *xbp-1(zc12)* mutant animals (Figure 5A), indicating that the UPR is not required for activation of p38 MAPK pathway in response to PFT. We extended this result using *ttm-2*, a downstream transcriptional target of the p38 MAPK pathway in response to Cry5B and a gene required for normal defense against Cry5B PFT [6]. Upregulation of *ttm-2* mRNA was dependent on the p38 MAPK pathway but not dependent on *xbp-1* (Figure 5F).

We next analyzed the reverse relationship between the *ire-1-xbp-1* and the p38 MAPK pathways, namely whether the p38 MAPK pathway is required for PFT-induced activation of the *ire-1-xbp-1* pathway. We find that activation of the *ire-1-xbp-1* pathway in response to PFT is dependent on the p38 MAPK pathway, namely on *sek-1*, the MAPK kinase (MAPKK) gene upstream of p38, and on *pmk-1*, the p38 MAPK downstream of *sek-1* (Figure 5). We find that increased splicing (activation) of *xbp-1* in response to Cry5B does not occur in *sek-1(km4)* MAPKK mutant animals (Figure 5B). Quantitatively, at the 3 h time point the spliced form of *xbp-1* is induced 1.9 fold in animals with an intact p38 MAPK pathway and depressed 1.8 fold in *sek-1(km4)* MAPKK mutant animals relative to untreated controls. However, *sek-1* is not absolutely required for splicing of *xbp-1* since, in response to tunicamycin, splicing of *xbp-1* is normal in *sek-1(km4)* mutant animals (Figure 5C). In agreement with these results, we find that *in vivo* activation of the downstream target of the *ire-1-xbp-1* pathway, *hsp-4::GFP*, by Cry5B within intestinal cells does not occur in *pmk-1(km25)* p38 MAPK mutant animals (Figure 5D), whereas activation of *hsp-4::GFP* by tunicamycin does occur normally in *pmk-1(km25)* mutant animals (Figure 5E).

To independently confirm and extend these results, we analyzed a different downstream target of the *ire-1-xbp-1* pathway. Using proteomics, we identified a protein, Y41C4A.11 (a homolog of the beta-prime subunit of the coatomer complex), that increased 4.6 fold in *C. elegans* animals exposed to Cry5B and whose increase was completely dependent on *xbp-1* (see Materials and Methods and Protocol S1). The gene encoding this protein was previously demonstrated to be transcriptionally regulated by tunicamycin in an *ire-1* and *xbp-1* dependent manner [12]. Using real time PCR, we find that both *hsp-4* mRNA and Y41C4A.11 mRNA are induced by either Cry5B or tunicamycin (Figure 5F). Consistent with activation of the *ire-1-xbp-1* pathway by p38 MAPK in response to PFT but not unfolded proteins, the full induction of both mRNAs by Cry5B, but not tunicamycin, is dependent on *sek-1* MAPKK. Interestingly, whereas induction of both mRNAs by Cry5B is lacking in *xbp-1(zc12)* mutant animals (confirming that activation of *hsp-4* and Y41C4A.11 by PFT is via the UPR), both mRNAs are still somewhat induced by Cry5B in a *sek-1(km4)* mutant, albeit at lower levels than in wild-type animals. These data suggest that some of the UPR-mediated transcriptional response is p38 pathway independent.

Based on these data, we predicted that animals mutant in the p38 pathway should be more sensitive to PFT than animals mutant in the UPR pathway. This hypothesis is based on the fact that the p38 pathway is upstream of the UPR, is required for full activation of the UPR in response to PFT, and is involved in UPR-independent PFT defense pathways (*e.g.*, *ttm-2*). Comparison of *sek-1(km4)* and *xbp-1(zc12)* mutant animals on Cry5B indicates *sek-1(km4)* animals are more severely intoxicated than *xbp-1(zc12)* animals at the same dose of Cry5B (Figure 5G). This conclusion was quantitatively confirmed by performing LC_50_ experiments on N2 and *sek-1(km4)* animals (Table 1). Whereas the LC_50_ of *xbp-1(zc12)* animals on Cry5B is 5.8 fold lower than N2, the LC_50_ of *sek-1(km4)* animals on Cry5B is 170 fold lower than N2.

## Discussion

Here we demonstrate that ER stress response pathways play a central but heretofore unknown role in innate defenses *in vivo*. Specifically, we find that bacterial pore-forming toxins (PFTs) activate the *ire-1-xbp-1* branch of the ER Unfolded Protein Response (UPR) in *C. elegans* and mammalian cells and that the *ire-1-xbp-1* and *atf-6*, but not the *pek-1*, branches of the UPR are important for *C. elegans* cellular defenses against a PFT since elimination of either of these two branches leads to hypersensitivity to the PFT Cry5B.

The ER stress response has been previously associated with pathogenic attack, mostly in the opposite direction shown here, *e.g.*, aiding viral replication and pathogenesis ([21] and references therein). In a few cases, the ER stress response has been linked with innate immunity since induction of ER stress can activate CREB-H, which in turn promotes the acute inflammatory response [22]. It has also been suggested that IRE-1 could influence immunity via its association with TRAF-2, which in turn can regulate NF-κB [23]. Data from studies in plants suggest that in response to pathogens, signals can be produced that lead to an “anticipatory” UPR to handle the massive synthesis of new secretory proteins required [24].

Here we definitively demonstrate a functional role of the UPR in defense against a pathogen *in vivo*. Loss of *xbp-1* leads to animals nearly 6 fold more susceptible to PFT whereas loss of *atf-6* leads to animals nearly 3 fold more susceptible.

Our data suggest that cells have adapted the UPR pathway for a *specific* response to PFTs in order to promote cellular defense against this common form of pathogenic attack. First, we found that loss of the *xbp-1* arm of the UPR does not lead to hypersensitivity to a heavy metal or hydrogen peroxide nor does loss of either *xbp-1* or *atf-6* lead to decreased protection against a bacterial pathogen that lacks a PFT. Second, the *ire-1-xbp-1* and *atf-6* arms of the UPR are involved in the defense but the *pek-1* arm is not. Third, the activation and function of the UPR in PFT defenses can be separated from the role of the UPR in dealing with unfolded proteins (here tested using the drug tunicamycin) in two ways: 1) the relative importance of the various arms of the UPR for defense against PFT is different than their importance for protection against unfolded proteins and 2) the activation of the *ire-1-xbp-1* pathway by PFT, but not unfolded proteins, requires p38 MAPK (see below).

**Figure 6:**
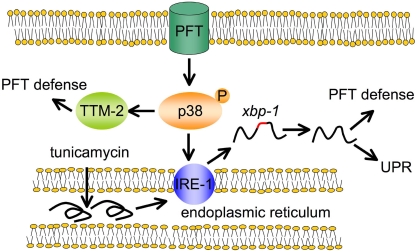
Schematic illustrating relationship between p38 MAPK, *ire-1-xbp-1*, and PFT defense pathways. PFTs at the cell surface of epithelial cells activate p38 MAPK that activates IRE-1 that induces splicing of *xbp-1*, which then turns on defense against PFTs. Residual activation of *xbp-1* targets in the absence of the p38 MAPK pathway suggests there might be p38-independent activation of the *ire-1-xbp-1* pathway in response to PFT as well (not shown). Independent of IRE-1 activation, p38 MAPK can also activate TTM-2 and other PFT defenses. Tunicamycin, which causes the accumulation of unfolded proteins in the ER, activates IRE-1 via a mechanism independent of the PFT and p38 MAPK.

A link between the p38 and UPR pathways has been shown in previous studies, although not with the level of functional relevance demonstrated here. Various arms of the UPR have been shown as both upstream or downstream of the p38 pathway, depending on the circumstances [25],[26],[27],[28],[29],[30]. The p38 pathway itself is implicated extensively in innate immune protection of many organisms against pathogens [31] and against PFTs in worms and mammals [6],[7]. Our data presented here for the first time functionally link the UPR to this major innate immune signal transduction pathway. Our findings on the activation and role of the UPR and p38 pathways in defense against PFT are summarized in Figure 6.

Why would induction of the ER stress response play a protective role against PFTs? It is possible that PFTs somehow lead to the accumulation of unfolded proteins in a cell. For example, PFTs are known to perturb calcium homeostasis and changes in calcium homeostasis are known to affect protein folding [32],[33]. In this model, cells would respond to the toxin via p38 MAPK and turn on the UPR to anticipate and ameliorate the detrimental effects of unfolded proteins. Arguing against this model, however, is our data showing that sensitivity of the three arms of the UPR to Cry5B is different than their sensitivity to a global unfolder of ER proteins, tunicamycin. A second model is that activation of the ER stress response by Cry5B in a p38 MAPK dependent manner may prepare the cell to handle an altered biosynthetic load in the ER to defend against a toxin. For example, transcriptional array analysis indicate that over 1000 genes are differentially regulated in *C. elegans* by Cry5B ingestion [6], which could in turn lead to significant changes in the protein load of the ER. A third model is based on the fact that activation of the *ire-1-xbp-1* pathway leads to increased phospholipid biogenesis [34]. It is possible that the defensive role of the *ire-1-xbp-1* pathway is to produce phospholipids that play a protective role against PFTs. Consistent with this, it has been shown that inhibiting the activation of SREBPs, the central regulators of membrane biogenesis, leads to hypersensitivity of mammalian cells to the PFT aerolysin [35].

In summary, we have identified specifically the *ire-1-xbp-1* and *atf-6* ER stress transducer pathways as components of cellular defenses against a PFT. While p38 MAPK was previously demonstrated to function in this regard [6], we have discovered a major and unexpected downstream target of this pathway for PFT defenses, namely the UPR. These results demonstrate the fundamental requirement for specific cell responses to bacterial PFTs and support the notion of intrinsic cellular defenses (or INCED, formerly, cellular non-immune defenses), a budding concept in immunity that emphasizes the intrinsic ability of epithelial cells to defend against bacterial toxins and the importance of these defenses as a supplement to the innate immune and adaptive immune systems [36]. Additionally, the differential importance of the three ER stress transducer pathways in response to Cry5B versus tunicamycin, the differential activation of *ire-1-xbp-1* by p38 MAPK in response to Cry5B versus tunicamycin, and the divergent pathways regulated by p38 MAPK in protective responses reveal how studying pathogenesis can uncover a wonderful complexity and new connections among intracellular pathways.

## Materials and Methods

### *C. elegans* Maintenance and Microscopy

*C. elegans* strains were maintained at 20°C on NG plates using *Escherichia coli* strain OP50 as the food source [37]. Strains used in this study were wild-type Bristol strain N2 [37], *atf-6(ok551)*, *glp-4(bn2)*, *ire-1(v33)*, *pek-1(ok275)*, *pmk-1(km25)*, *sek-1(km4)*, SJ4005 (zcIs4 [hsp-4::GFP]) and *xbp-1(zc12)*. *atf-6(ok551)* and *pek-1(ok275)* were each outcrossed a total of 6 times. SJ4005 was outcrossed an additional 4 times as it had been outcrossed twice upon receipt from the *Caenorhabditis* Genetics Center. *xbp-1(zc12)* was created by outcrossing strain SJ17 *(xbp-1(zc12)*; zcIs4 [hsp-4::GFP]) four times and removing the integrated hsp-4::GFP during the outcrosses. Images were acquired with an Olympus BX60 microscope with the 10× objective linked to a 0.5× camera mount and a DVC camera. Worms were placed on 2% agarose pads containing 0.1% sodium azide for photography.

### Toxicity Assays

All assays were performed at 20°C unless indicated elsewhere. Qualitative toxicity assays based on visual comparison of worm intoxication were performed on plates with *E. coli*-expressed Cry5B as described [6],[38]. Beginning with the 4^th^ larval (L4) stage worms, worms were fed for 48 hours either on control plates with *E. coli* JM103 that did not express Cry5B (empty vector) or plates prepared with *E. coli* JM103 expressing Cry5B diluted 1∶3 with empty vector transformed JM103. This amount of Cry5B (25%) mildly intoxicates wild-type *C. elegans*, which allows for identification of strains that are hypersensitive to Cry5B as these strains will be more severely intoxicated than wild type. Quantitative lethal concentration assays were performed as described [38] except the worms were scored after 8 days for Cry5B, CuSO_4_, and tunicamycin. Lethal concentration assays with H_2_O_2_ did not include *E. coli* or 5-fluoro-2′-deoxy-uridine, and worms were scored after 4 hours. Concentrations of each toxin were set-up in triplicate for each assay, and each assay was performed independently three times. Purified Cry5B was prepared as described [39] and dissolved in 20 mM HEPES (pH 8.0) prior to use. Approximately 1500 worms were scored for each strain in the calculation of the LC_50_ values for each toxin. For tunicamycin assays, the set up was identical to the lethality assay with Cry5B. For the developmental inhibition assay, Cry5B plates were prepared as described [6],[38]. Approximately 100 L1 stage worms (from bleached embryos hatched off overnight) were placed on each plate (60 mm) and the number of worms at the L4 or adult stage 3 days later was determined. This assay was performed independently three times. The *P. aeruginosa* lifespan assay was performed on slow-killing plates as described [40], with the following modifications: PA14 was cultured overnight in tryptic soy broth instead of King's broth and then spread on slow-killing plates complemented with 75 uM µM 5-fluoro-2′-deoxy-uridine. The experiment was performed three times with approximately 100–150 worms total per strain, at 20°C. To determine if there was rescue of the hypersensitivity phenotype in the intestinal-specific promoter studies, 25% *E. coli*-expressing Cry5B plates were used to compare Cry5B sensitivities of wild-type N2, *xbp-1(zc12)*, and *xbp-1(zc12)* that were transformed with constructs to express either green fluorescent protein (GFP) or *xbp-1* mRNA within intestinal cells using the *app-1* promoter (plasmids are described in Protocol S1). Transgenic L4 stage worms were placed on the 25% *E. coli* expressing Cry5B plates and their health status was assessed 72 hours later. Specifically, the relative health of each worm was determined qualitatively by comparing body size, darkness of the intestine as an indicator of feeding, and activity, including whether the worm demonstrated spontaneous movement. For scoring of the transgenic worms, comparisons were made using both N2 as a reference for healthy worms, as they demonstrated dark intestines and continuous spontaneous movement, and *xbp-1 (zc12)* as a reference for intoxicated worms that had pale intestines and demonstrated rare or no spontaneous movement.

### *xbp-1* splicing and real time PCR

The *glp-4(bn2)* strain was used for these experiments (including the double mutants *glp-4(bn2)*;*xbp-1(zc12)* an *glp-4(bn2)*; *sek-1(km4)*) since it has a greatly reduced number of germ cells when grown at 20°C. This helps remove the background of macromolecules not isolated from the intestine. The response to Cry5B is not altered in this strain compared to wild type [6]. Primers used for these experiments are described in Protocol S1. Approximately 15,000 L4 stage worms were used per 100 mm dish for each treatment group. Worms were exposed to Cry5B for the indicated period of time on either *E. coli* JM103 containing empty vector or *E. coli* JM103 expressing Cry5B as described [6],[38]. After exposure to each treatment, worms were rinsed from plates with water, centrifuged at 500 g for 45 seconds, and washed two additional times with water. RNA was prepared from worms using TRIZOL (Invitrogen) and further purified with RNeasy columns (Qiagen). cDNA was prepared by reverse transcription using oligo-dT. Standard PCR was used to detect *xbp-1* splicing, and products were analyzed on 2% agarose gel. Unspliced *xbp-1* transcript is 220 nucleotides and spliced transcript is 197 nucleotides. To quantitate the amount of *xbp-1* splicing, loading was normalized by quantitating cDNA levels using real time PCR and *eft-2* primers [6]. Equal amounts of cDNA were used for the *xbp-1* splicing PCR experiments and 10 microliters of each reaction were loaded onto a 2% agaose gel and stained with ethidium bromide. NIH ImageJ was then used to quantitate the intensities of *xbp-1* spliced forms in Cry5B treated samples relative to untreated samples at the same time point.

Real time PCR was performed on an ABI 7000 Instrument using SYBR Green detection (Applied Biosystems). *eft-2* was used as the real time PCR normalization control [6]. Experiments with Cry5B used either a control plate (*E. coli* not expressing Cry5B) or a Cry5B plate on which 100% of the *E. coli* expressed Cry5B. Tunicamycin experiments used *E. coli* OP50 as a food source and either DMSO as the control or tunicamycin at 2 µg/mL incorporated into the plates. Three independent experiments for the splicing and real time PCR were performed for each treatment.

### Mammalian XBP-1 immunoblotting

HeLa cells were cultured in MEM media supplemented with 10% fetal calf serum, 1% penicillin-streptomycin, 1% glutamine and 1% non-essential amino acids, in a humidified incubator with 5% CO_2_ at 37°C. Aerolysin was purified as described [41]. Cells were continuously treated with 2 ng/mL (0.02 nM) of proaerolysin. At different time points, cells were washed with PBS and lysed at 4°C in 0.25 M sucrose supplemented with proteases inhibitor (Roche, Germany) using a needle. The whole cell extracts were subjected to SDS-PAGE and Western blotting. XBP1 (R-14) antibody was from Santa Cruz Inc. Band intensities were quantified, after background removal, using ImageJ software (NIH). The loading in each lane was normalized relative to the intensity of a nonspecific antibody-reacting band on the blot.

### p38 MAPK immunoblotting

Approximately 750 L1 stage worms were grown in a single well of a 48 well plate containing 150 µL S media [42] and *E. coli* OP50. When worms had reached the L4 to young adult stage, glucose was added to 100 mM and either 20 mM HEPES (pH 8.0) or Cry5B dissolved in 20 mM HEPES (pH 8.0) to give a final concentration of 100 µg/mL was added. After one hour, worms were removed, centrifuged, and 175 µL of media was removed. Twenty five µL of 2× sodium dodecyl sulfate loading buffer was added, and worms were boiled for 5 minutes. Ten microliters of lysate were used for immunoblotting. Monoclonal antibody to phospho P38 MAPK (Cell Signaling Technology cat. no. 9215) was used at 1∶300 and monoclonal antibody to α-tubulin (Sigma-Aldrich cat. no. T6199) was used at 1∶4000.

### Proteomics

L4 stage *glp-4(bn2)* and *glp-4(bn2)*;*xbp-1(zc12)* worms were used for this experiment. Approximately 80,000 worms of each strain were used for both control and Cry5B treatments. Control plates consisted of 100 mm plates spread with *E. coli* that did not express Cry5B, while Cry5B treatments consisted of plates in which 100% of the *E. coli* expressed Cry5B. Approximately 20,000 worms were used per plate. Worms were fed on the bacteria for 6 hours at 20°C. For details of mass spectrometry, please see Protocol S1.

### Data analysis

All experiments were performed a minimum of three times. LC_50_ values were determined by PROBIT analysis [43]. The lethal concentration assays are represented graphically using nonlinear regression performed with the software GraphPad Prism. Statistical analysis between two values was compared with a paired t-test. Statistical analysis among three or more values was compared with matched one way ANOVA using the Tukey post test. Lifespan data was analyzed with Kaplan-Meier survival curves. Statistical significance was set at p<0.05.

## Supporting Information

Protocol S1 (0.03 MB DOC)
