# Supplementary material for: Activation of the Unfolded Protein Response Is Required for Defenses against Bacterial Pore-Forming Toxin In Vivo
Source: PLoS Pathog. 2008 Oct 10;4(10):e1000176. doi: 10.1371/journal.ppat.1000176 (PMC2553261; doi:10.1371/journal.ppat.1000176)
Supplement: Protocol S1 — (0.03 MB DOC) [file ppat.1000176.s001.doc]

# Supporting information

# Oligonucleotides, plasmids and transgenic worms

For *xbp-1* mRNA splicing, the following oligonucleotides were used: CGTCGTCTACGAAGAAGAAGTCGTC and GATGATAGTTAGATACATATCCACACTG. For real-time PCR, the following oligonucleotides were used, *eft-2*: TCGAAATTCAATGCCCAGAAG and CTCCTCGAAAACGTGTCCTCTT; *hsp-4:* GGATCAACCAGAATTCCAAAGG and TCAGGGTTGATTCCACGAGAT; Y41C4A.11: TGGTCAAGGCTATGGAGGTC and ATTTGCTGATCATCGGAAGC; *ttm-2*: GCTGGACAAAATACGATTCACA and TCCATGAAGTCTGCCTTCTACA.

The *app-1::GFP* and *app-1::xbp-1* plasmids were created using pPD95.75 (Addgene plasmid 1494, please see <http://www.addgene.org/pgvec1> for details of this plasmid). A 1231 nucleotide promoter fragment of *app-1* was generated by PCR using wild-type N2 genomic DNA with the oligonucleotides GGTGCATGCCACCATCGGCGCATAAGGTATAG and GGTGCATGCCACTCCAATTCTGCCCGATCTG and ligated into pPD95.75 at an SphI site to create *app-1::GFP*. To create *app-1::xbp-1*, pPD95.75 was initially digested with AgeI and EcoRI to remove the GFP gene and then ligated. The coding region of the *xbp-1* gene was generated by PCR with the oligonucleotides GTTTCTAGACCCCATATTCGCTACACTG and TTTCCCGGGCGAGGGAAATTCACTAGGACAC from wild-type N2 genomic DNA. The *xbp-1* gene was inserted at XbaI and SmaI sites into pPD95.75 lacking GFP. The *app-1* promoter was then removed from plasmid *app-1::GFP* at HindIII and HincII sites and inserted into the *xbp-1* containing plasmid at these same restriction sites to create the final *app-1::xbp-1* plasmid. There are 3 introns contained within this *xbp-1*gene, including the 23 base pair intron that is spliced by activated *ire-1*. The correct sequences of the *app-1* promoter and *xbp-1* gene were verified by DNA sequencing.

To create transgenic lines of *xbp-1 (zc12)*, adults of this strain were injected with either: 1) *app-1::GFP* plasmid (100 ng/µL) and *sur-5::GFP* plasmid [1] (50 ng/µL); or 2) *app-1::xbp-1* plasmid (100 ng/µL) and *sur-5::GFP* plasmid (50 ng/µL). First generation progeny from the injected worms that expressed GFP were maintained as separate lines for each transgenic construct. Progeny from the distinct lines were tested in the *xbp-1* intestinal rescue of Cry5B toxicity assay as described in the MATERIALS and METHODS.

# Mass spectrometry

Following feeding on control or Cry5B expressing *E. coli*, the worms were rinsed from each plate with H_2_O. Worms were centrifuged at 500g for 45 seconds, the supernatant was aspirated and the worms were washed in H_2_O and centrifuged as before. The water wash was then repeated. To each worm pellet was added 2% RapiGest SF (Waters) in 10 mM Tris-Cl (pH 8.0), 100 mM NaCl and 1 mM EDTA. The worms were frozen in liquid nitrogen and then exposed to three freeze thaw cycles on dry ice. Worms were sonicated three times for 20 seconds each at 5 Watts. The solution was centrifuged for three minutes at 500g, and the supernatant was transferred and processed for mass spectrometry analysis as follows.

Fifty µL of each sample was diluted 10 times in 25 mM Hepes buffer (pH7.2). The proteins were reduced and alkylated using 1 mM Tris(2-carboxyethyl)phosphine (Fisher, AC36383) at 95°C for 5 minutes and 2.5 mM iodoacetamide (Fisher, AC12227) at 37°C in dark for 15 minutes, respectively. The proteins were digested with 1:50 trypsin (Roche, 03 708 969 001) overnight. Fifty µg of digested peptides of P0 and P22 sample was labeled by iTRAQ reagents (114-*glp-4(bn2),* control; 115- *glp-4(bn2),* Cry5B; 116- *glp-4(bn2);xbp-1(zc12),* control; 117- *glp-4(bn2);xbp-1(zc12),* Cry5B), respectively. The labeled peptides were mixed together. 1% TFA was added to the mixture to precipitate RapiGest (pH 1.4). Sample was incubated at 4^o^C overnight and then centrifuged at 16.1Kg for 15 minutes. Supernatant was collected and passed through a 0.22 µM spin filter. The cleared solution was subject to Nano-LC-MS/MS analysis.

Automated 2D nanoflow LC-MS/MS analysis was performed using LTQ tandem mass spectrometer (Thermo Electron Corporation, San Jose, CA) employing automated data-dependent acquisition. An Agilent 1100 HPLC system (Agilent Technologies, Wilmington, DE) was used to deliver a flow rate of 300 nL min^–1^ to the mass spectrometer through a splitter. Chromatographic separation was accomplished using a 3 phase capillary column. Using an in-house constructed pressure cell, 5 µm Zorbax SB-C18 (Agilent) packing material was packed into a fused silica capillary tubing (200 µm ID, 360 µm OD, 20 cm long) to form the first dimension RP column (RP1). A similar column (200 µm ID, 5 cm long) packed with 5 µm PolySulfoethyl (PolyLC) packing material was used as the SCX column. A zero dead volume 1 µm filter (Upchurch, M548) was attached to the exit of each column for column packing and connecting. A fused silica capillary (100 µm ID, 360 µm OD, 20 cm long) packed with 5µm Zorbax SB-C18 (Agilent) packing material was used as the analytical column (RP2). One end of the fused silica tubing was pulled to a sharp tip with the ID smaller than 1 µm using a laser puller (Sutter P-2000) as the electro-spray tip. The peptide mixtures were loaded onto the RP1 column using the same in-house pressure cell. Peptides were first eluted from RP1 column to SCX column using a 0 to 80% acetonitrile gradient for 150 minutes. Then the peptides were fractionated by the SCX column using a series of 27 salt gradients (20mM, 30mM, 35mM, 40mM, 42mM, 45mM, 47mM, 50mM, 52mM, 55mM, 57mM, 60mM, 62mM, 65mM, 67mM, 70mM, 72mM, 75mM, 77mM, 80mM, 85mM, 90mM, 95mM, 100mM, 120mM, 150mM, 180mM, 200mM, 500mM, 1M ammonium acetate for 20 minutes), followed by high resolution reverse phase separation using an acetronitrile gradient of 0 to 80% for 120 minutes.

The full MS scan range of 400-2000 m/z was divided into 3 smaller scan ranges (400-800, 800-1050, 1050-2000) to improve the dynamic range. Both CID (Collision Induced Dissociation) and PQD (Pulsed-Q Dissociation) scans of the same parent ion were collected for protein identification and quantitation. Each MS scan was followed by 4 pairs of CID-PQD MS/MS scans of the most intense ions from the parent MS scan. A dynamic exclusion of 1 minute was used to improve the duty cycle of MS/MS scans. About 20,000 MS/MS spectra were collected for each sample.

The raw data was extracted and searched using Spectrum Mill (Agilnet, version A.03.02). The CID and PQD scans from the same parent ion were merged together. MS/MS spectra with a sequence tag length of 1 or less were considered as poor spectra and discarded. The rest of the MS/MS spectra were searched against the NCBI (National Center for Biotechnology Information) RefSeq protein database (version 18, July 2006) limited to *C. elegans* (22,856 sequences). The enzyme parameter was limited to full tryptic peptides with a maximum miscleavage of 1. All other search parameters were set to SpectrumMill’s default settings (carbamidomethylation of cysteines, iTRAQ modification, +/- 2.5 Da for precursor ions, +/- 0.7 Da for fragment ions, and a minimum matched peak intensity of 50%). A concatenated forward-reverse database was constructed to calculate the *in-situ* false discovery rate (FDR). A total of 3,978 RefSeq proteins from the forward database were identified, while 156 proteins (4% protein FDR) from the reverse database were identified.

Relative protein quantitation was performed by calculating the iTRAQ reported ion intensity ratios. Protein iTRAQ intensities were calculated by summing the peptide iTRAQ intensities from each protein group. Peptides shared among different protein groups were removed before quantitation.

1. Yochem J, Gu T, Han M (1998) A new marker for mosaic analysis i*n Caenorhabditis elega*ns indicates a fusion between hyp6 and hyp7, two major components of the hypodermis. Genetics 149: 1323-1334.
